# The *Solanum lycopersicum* WRKY3 Transcription Factor SlWRKY3 Is Involved in Salt Stress Tolerance in Tomato

**DOI:** 10.3389/fpls.2017.01343

**Published:** 2017-07-31

**Authors:** Imène Hichri, Yordan Muhovski, Eva Žižková, Petre I. Dobrev, Emna Gharbi, Jose M. Franco-Zorrilla, Irene Lopez-Vidriero, Roberto Solano, André Clippe, Abdelmounaim Errachid, Vaclav Motyka, Stanley Lutts

**Affiliations:** ^1^Groupe de Recherche en Physiologie Végétale, Earth and Life Institute - Agronomy, Université Catholique de Louvain Louvain-la-Neuve, Belgium; ^2^Département Sciences du Vivant, Centre Wallon de Recherches Agronomiques Gembloux, Belgium; ^3^Institute of Experimental Botany, Academy of Sciences of the Czech Republic Prague, Czechia; ^4^Departamento de Genética Molecular de Plantas, Centro Nacional de Biotecnología-Consejo Superior de Investigaciones Científicas, Campus Universidad Autónoma Madrid, Spain; ^5^Institut des Sciences de la Vie, Université Catholique de Louvain Louvain-la-Neuve, Belgium

**Keywords:** *Solanum lycopersicum*, SlWRKY3, transcription factor, salinity tolerance, plant physiology

## Abstract

Salinity threatens productivity of economically important crops such as tomato (*Solanum lycopersicum* L.). WRKY transcription factors appear, from a growing body of knowledge, as important regulators of abiotic stresses tolerance. Tomato SlWRKY3 is a nuclear protein binding to the consensus CGTTGACC/T W box. *SlWRKY3* is preferentially expressed in aged organs, and is rapidly induced by NaCl, KCl, and drought. In addition, *SlWRKY3* responds to salicylic acid, and *35S*::*SlWRKY3* tomatoes showed under salt treatment reduced contents of salicylic acid. In tomato, overexpression of *SlWRKY3* impacted multiple aspects of salinity tolerance. Indeed, salinized (125 mM NaCl, 20 days) *35S::SlWRKY3* tomato plants displayed reduced oxidative stress and proline contents compared to WT. Physiological parameters related to plant growth (shoot and root biomass) and photosynthesis (stomatal conductance and chlorophyll *a* content) were retained in transgenic plants, together with lower Na^+^ contents in leaves, and higher accumulation of K^+^ and Ca^2+^. Microarray analysis confirmed that many stress-related genes were already up-regulated in transgenic tomatoes under optimal conditions of growth, including genes coding for antioxidant enzymes, ion and water transporters, or plant defense proteins. Together, these results indicate that SlWRKY3 is an important regulator of salinity tolerance in tomato.

## Introduction

Excessive salt accumulation represents a challenge for the cultivation of economically important crops such as tomato (*Solanum lycopersicum* L.). Cultivated tomato is overall considered as moderately (∼70 mM NaCl) tolerant to salinity (Pérez-Alfocea et al., 1996). Salinity affects numerous traits of plant growth (biomass formation, root/shoot ratio, ..), biochemistry (photosynthesis, hormonal and nutritional imbalance, …) and physiology, and consequently final yield (Albacete et al., 2008). Important efforts are devoted to deciphering the complex aspects of salinity response and tolerance in tomato (Cuartero et al., 2006).

Transcription factors (TFs), including WRKY-type proteins, regulate a plethora of downstream stress-related genes, leading to biochemical and physiological modifications necessary for plant adaptation (Ouyang et al., 2007; Phukan et al., 2016). WRKY family’s name is attributed to the conserved heptapeptide WRKYGQK motif and its variants, positioned at the N-terminal end of an approximately 60 amino acids DNA binding region. The so called WRKY domain constitutes the hallmark of the family, and classically defines four-stranded β sheets including an atypical C2H2 or C2HC zinc-finger structure following the WRKY stretch of residues (Rushton et al., 2010; Yamasaki et al., 2012). Consistent with the number of WRKY domains, the structure of the zinc-finger motif, in addition to the presence of extra conserved motifs such as leucine zipper or serine-threonine rich regions, WRKY are principally divided into three groups (I, II, and III), the second one being further split into three subgroups (IIa + IIb, IIc, IId + IIe) (Ülker and Somssich, 2004; Rushton et al., 2010; Rinerson et al., 2015a).

WRKY proteins regulate multiple processes related to plant development and reproduction (Zhang et al., 2011; Guo et al., 2015; Yang et al., 2016), phenolic compounds biosynthesis (Schluttenhofer and Yuan, 2015), hormonal signaling (Yu et al., 2010; Zhang et al., 2015), or senescence (Rinerson et al., 2015b). However, fundamental roles of WRKY regulators reside in their involvement in plant defense signaling in response to pathogens, nematodes or herbivores, and from a growing body of evidence, in tolerance to abiotic stresses (Rushton et al., 2010; Chen et al., 2012; Gong et al., 2015; Banerjee and Roychoudhury, 2015). In soybean (*Glycine max*), 25 out of 64 tested *WRKY* genes respond to high salinity, drought or cold treatments (Zhou et al., 2008; Song et al., 2016), and comparable results have been reported in Arabidopsis (Jiang and Deyholos, 2006), *Gossypium aridum* (Fan et al., 2015), wheat (*Triticum aestivum* L.) or rice, among others (Berri et al., 2009; Niu et al., 2012).

Functional characterization of WRKYs involved in salt stress response have been conducted in different plant species, and highlighted the involvement of WRKY regulators in oxidative stress management (Liu et al., 2013; Wang et al., 2013; Zheng et al., 2013; Shi et al., 2014; Wang X. et al., 2015; Agarwal et al., 2016). Wheat TaWRKY2, TaWRKY19, TaWRKY44, and TaWRKY93 improve tolerance to salinity and drought by enhancing osmoprotectants (proline and soluble sugars) accumulation and improving oxidative stress management (Niu et al., 2012; Qin et al., 2015; Wang X. et al., 2015). Cotton (*Gossypium hirsutum*) GhWRKY39-1, GHWRKY34, and GhWRKY41 enhance plant germination and growth in presence of salt, reduce sodium and ROS accumulation (Shi et al., 2014; Chu et al., 2015; Zhou et al., 2015), and similar mechanisms of tolerance have been reported for chrysanthemum (*Dendranthema grandiflorum*) DgWRKY3 (Liu et al., 2013), *Gossypium aridum* GarWRKY17 and GarWRKY104 (Fan et al., 2015), or for a *Jatropha curcas* WRKY (Agarwal et al., 2016). *Tamarix hispida* ThWRKY4 improves Arabidopsis plants germination and growth in presence of salt, and prevents chlorophyll degradation, together with oxidative stress control (Zheng et al., 2013). Additional regulators of salt stress response have been described, and include Arabidopsis AtWRKY25, AtWRKY33 (Jiang and Deyholos, 2009) and AtWRKY8 (Hu et al., 2013), together with AtWRKY46 (Ding et al., 2014).

Very few WRKY (namely SlWKRY and SpWRKY1) induced by salt and drought has been hitherto partially characterized in tomato, and their constitutive expression in tobacco resulted in higher tolerance to these stresses by improving plant growth and reducing oxidative stress (Li et al., 2012, 2015). Herein we identified *SlWRKY3* [which actually corresponds to *SlWRKY81* of Huang et al. (2012) classification], which has been reported as up-regulated by salinity and different pathogens (Huang et al., 2012). This study describes the functional characterization of SlWRKY3 in response to salinity. *SlWRKY3* is induced by diverse osmotic stresses, in addition to salicylic acid (SA). Constitutive overexpression of *SlWRKY3* in tomato enhanced the expression of genes involved in abiotic stress management, ion and water transport, and cellular detoxification. It improved plant tolerance to salinity, following physiological, biochemical and hormonal modifications. Altogether, these results indicate that SlWRKY3 is a novel regulator of tolerance to osmotic stresses in tomato.

## Materials and Methods

### Plant Material and Osmotic Stresses/Hormonal Treatments Assays

Tomato (*S. lycopersicum* cv Ailsa Craig) seedlings (3 weeks old) were cultivated in 52 L tanks containing aerated half-strength Hoagland nutrient solution as described in Hichri et al. (2014). Plants were grown in a phytotron at 24°C/22°C under a 16 h day/8 h night photoperiod (230 μmol.m^-2^.s^-1^). Arabidopsis (*Arabidopsis thaliana* cv Columbia) were grown in soil at 20°C/19°C under a 16 h day/8 h night photoperiod (190 μmol.m^-2^.s^-1^).

For *in vitro* culture, tomato and Arabidopsis seeds were surface sterilized (Hichri et al., 2014). Transgenic tomato seeds were germinated on MS medium containing 100 mg/L then 200 mg/L kanamycin for seedlings, at 24°C under a 16 h day/8 h night regime. Arabidopsis seeds were cold-treated (48 h, 4°C), and subsequently plated on half strength MS medium, supplemented or not with 35 mg/L kanamycin.

For salinity treatment assays, salt (125 mM NaCl) was added to the culture tank. To conduct multiple osmotic stresses (150 mM NaCl, 150 mM KCl or drought by air drying at 21°C) and hormonal assays during 3 h (for both types of treatments), plants were grown in smaller tanks with a capacity of 4.5 L.

Zeatin (mixed isomers including approximately 80% *trans*-zeatin), abscisic acid, salicylic acid, and indole acetic acid were used at a final concentration of 10 μM.

### RNA Extraction and Quantitative RT-PCR Analysis

Tomato total RNA extraction, DNase I treatment, RNA purification, reverse-transcription and quantitative RT-PCR (qRT-PCR) analyses were conducted as described in Hichri et al. (2014). Sequences of primers used for qRT-PCR analysis are available in Supplementary Table S1.

Data were normalized according to the *SlGAPDH, SlActin*, and *SlEF1*α housekeeping genes expression levels. Normalized expression was calculated using the Gene Expression Analysis for iCycleiQ_ Real Time PCR Detection System software from Bio-Rad with a method derived from the algorithms outlined by Vandesompele et al. (2002).

### Constructs and Plant Transformation

*SlWRKY3* cDNA was amplified by PCR with the *Pfu* DNA polymerase (Promega) following the manufacturer’s instructions. *SlWRKY3* cDNA was subcloned into pGEM-T-easy vector (Promega). Subsequently, *SlWRKY3* open reading frame was cloned into the pDONR221 entry vector (Gateway^TM^ Technology, Invitrogen) by a BP recombination reaction prior to sequencing. *SlWRKY3* was consequently transferred to the pK7WG2D binary vector (Karimi et al., 2002) by a LR recombination reaction (Gateway^TM^ Technology, Invitrogen). The recombinant plasmid was introduced into *Agrobacterium tumefaciens* strain GV3101 for Arabidopsis transformation and LBA4404 (Clontech) for tomato transformation.

Arabidopsis plants were transformed by the floral dip method (Clough and Bent, 1998) and analyses conducted on self-fertilized F3 homozygous transgenic lines. Tomato stable transformation was adapted from Ellul et al. (2003), and analyses conducted on F3 homozygous lines. All the transgenic lines described are available in S. Lutts laboratory.

To determine SlWRKY3 subcellular localization, *SlWRKY3* ORF was introduced in frame into the pYFP-attR vector (Subramanian et al., 2006) which allows transient expression of the protein via N-terminal fusion to the Yellow Fluorescent Protein. Isolation, purification and PEG-mediated transformation of tomato protoplasts starting from young leaves of *in vitro* grown plants have been conducted as previously described (Hichri et al., 2010). YFP, YFP–SlWRKY3 fusion protein, and chlorophyll fluorescence were visualized using a Zeiss 710 confocal microscope (Carl Zeiss, Jena, Germany) using a plan-apochromat 63×/1.40 objective. Images were analyzed with the Zen Software (Zeiss).

### Constructs and Yeast Transformation

For yeast auto-activation assay, *SlWRKY3* ORF was cloned between *Eco*RI and *Bam*HI restriction sites into the pGBKT7 vector (BD Bioscience) by in frame fusion to the GAL4 DNA Binding Domain (DBD) coding region, under control of the *ALCOHOL DEHYDROGENASE1* (*ADH1*) promoter. pGBKT7 carries the *TRYPTOPHAN1* (*TRP1*) nutritional marker. Recombinant plasmids were introduced into the Y8930 yeast strain, presenting the *ADE2* and *HIS3* reporter genes.

### Protein Purification and Protein Binding Microarray

*SlWRKY3* was cloned into the pMAL-c2 expression vector (New England Biolabs) between *Bam*HI and *Pst*I restriction sites, allowing in frame fusion of SlWRKY3 with the maltose binding protein (MBP) coding sequence. Construct was introduced into the *Escherichia coli* BL21 strain. *SlWRKY3* expression was induced with 1 mM isopropyl β-D-1-thiogalactopyranoside, during 5 h at 37°C. Protein binding array experiments were conducted as described in Hichri et al. (2014).

### Osmotic Potential, Malonyldialdehyde and Proline Extraction and Quantification, Cell Membrane Stability and Cell Viability

Osmotic potential was measured on Leaf 5 (fifth fully expanded leaf from the bottom of the plant) according to Zhu et al. (2001) using a vapor pressure osmometer (Wescor 5500). Malonyldialdehyde was extracted from Leaf 5 by the thiobarbituric acid reaction as reported in Hichri et al. (2014). Proline was quantified spectrophotometrically on Leaf 5 using the ninhydrin method according to Bates et al. (1973).

Cell membrane stability was assessed on the basis of the leakage of UV absorbing substances (Redmann et al., 1986). The relative leakage ratio was quantified on Leaf 5 after incubation of leaf segments in the presence of either deionized water or 100 mM NaCl (Lutts et al., 1996). Cell viability was determined through reduction of 2,3,5-triphenyltetrazolium chloride (TTC) from samples incubated at 30°C in darkness in tubes containing 0.5% (v/v) TTC in 50 mM K_2_HPO_4_, pH 7.0 for 15 h (Lutts et al., 2004). The produced formazan was extracted with ethanol 94% (v/v) at 80°C during 5 min and quantified spectrophotometrically at 487 nm.

### Chlorophyll, Mineral and Hormones Extraction and Quantification, Stomatal Conductance

Relative water content was calculated from fresh, turgid (after 5 h rehydration in 30 mL sealed vials containing deionised water, in darkness at 4°C) and dry weight of leaf segments collected on Leaf 4. Chlorophyll was extracted from Leaf 5 using acetone 80% (v/v) and quantified spectrophotometrically (Lutts et al., 1996).

For mineral extraction (Leaf 4), samples were oven-dried during 3 days at 70°C, and 50–100 mg dry weight of plant samples were digested in perchloric acid/nitric acid (3:1, v/v) mixture (Hichri et al., 2014). Sodium, potassium and calcium ion concentrations were determined using an atomic absorption spectrometer (Thermo Scientific ICE 3300; Waltham, US-MA). All measurements were performed in triplicate.

Hormones were extracted as detailed in Hichri et al. (2014).

The stomatal conductance (*g*_s_) was measured on Leaf 4 using an AP4 system (Delta-T Devices; Cambridge, United Kingdom) between 2 and 4 pm.

### Microarray Analysis

Total RNA was extracted from four tomato seedlings (whole seedlings including roots, 4 weeks-old) of WT and W3 line transgenic plants grown *in vitro* on MS medium and purity and yield were assessed as described before (Hichri et al., 2014). RNA extraction, fluorescence labeling, statistical analysis and annotation of genes for each technical repetition (RNA extraction) was conducted as described in Hichri et al. (2014). Data were submitted to the GEO repository under GSE74504 reference.

### Statistical Analysis

Statistical analyses were conducted with SAS software (SAS System for Windows version 9.1, SAS Institute Inc., Carry, NC, United States). An analysis of variance (ANOVA) using the mean discrimination was performed on all data set using the Student–Newman–Keuls test at the 5% level.

### Accession Numbers

Accession numbers are the following: SlWRKY3 (ADZ15316), VpWRKY1 (ACY69975), GarWRKY5 (AIY62459), JcWRKY56 (AGQ04250), FcWRKY70 (AKA59519), StWRKY6 (ABU49725), NtWRKY4 (AAF61864), SmWRKY7 (AKA27873), AtWRKY70 (NP_191199), AtWRKY62 (NP_195810) and AtWRKY38 (NP_197649), BnWRKY70 (ACQ76810), NcWRKY53 (ABN79278), OsWRKY45 (DAA05110), OsWRKY100 (NP_001056863), TaWRKY45 (ABO15542).

## Results

### SlWRKY3 Encodes a WRKY Transcriptional Activator

A previous study allowed the identification of tomato TFs putatively involved in salinity tolerance, including an unknown WRKY candidate (initial accession DY523772) (Ouyang et al., 2007). Its 972 bp sequence including an open reading frame of 876 bp was amplified by PCR. The cloned cDNA was initially termed *SlWRKY3* (GenBank accession HQ706095) and actually corresponds to *SlWRKY81* (Solyc09g015770.2.1) of Huang et al. (2012) classification. *SlWRKY3* belongs to Group III, comprising 11 members in tomato (Huang et al., 2012).

**FIGURE 1:**
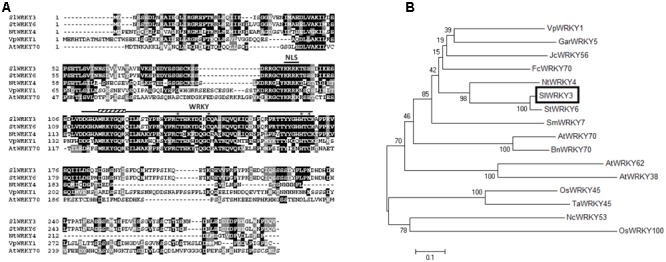
Identification of SlWRKY3. **(A)** Comparison of SlWRKY3 sequence with StWRKY6, NtWRKY4, VpWRKY1, and AtWRKY70. Identical residues are colored in black, and conserved residues in dark gray. The WRKY domain and the putative nuclear localization site are labeled. The Cys and His residues forming the zinc-finger motif are indicated by stars. **(B)** Phylogenetic analysis of SlWRKY3 and related proteins: StWRKY6, NtWRKY4, GarWRKY5, JcWRKY56, FcWRKY70, SmWRKY7, VpWRKY1, AtWRKY70, AtWRKY62 and AtWRKY38, NcWRKY53, BnWRKY70, OsWRKY45, OsWRKY100, and TaWRKY45. The phylogenetic tree was constructed according to the neighbor-joining method, using MEGA6 (Tamura et al., 2013). The percentage of reliability of each branch point of the rooted tree, as assessed by the analysis of 1000 trees (bootstrap replicates), is shown on the branch stem.

*SlWRKY3* is predicted to encode a 291 amino acids (aa) protein, with a molecular weight of 33 kDa. Analysis of SlWRKY3 sequence indicated the existence of a putative basic monopartite NLS KRRK, between aa 93 and 96 (**Figure 1A**). SlWRKY3 bears a WRKY domain of 59 residues starting at aa 111, including the WRKYGQK conserved stretch as labeled in **Figure 1A**, and followed by an atypical C_2_HC zinc-finger motif, **C**-X_7_-**C**-X_23_-**H**T**C** (from Cys134 to Cys169, **Figure 1A**). Besides, SlWRKY3 carries the conserved motif 17 of unknown function in its N-terminal end, shared between members of Group III and Group II-b of the tomato WRKY proteins family (Huang et al., 2012), as well as two serine rich regions (residues 79–86 and 224–227) (**Figure 1A**).

A phylogenetic tree of SlWRKY3 and 15 additional WRKY proteins belonging to the sub-group III was constructed by means of the neighbor-joining method using full-length aa sequences (**Figure 1B**). SlWRKY3 clusters with the potato (*S. tuberosum*) StWRKY6 and tobacco (*Nicotiana tabacum*) NtWRKY4, both induced by pathogens and/or SA (Chen and Chen, 2000) (**Figure 1B**). It also clusters with *Fortunella crassifolia* FcWRKY70 (Gong et al., 2015), JcWRKY56 (Xiong et al., 2013), *G. aridum* GarWRKY5 (Fan et al., 2015), and *Vitis pseudoreticulata* VpWRKY1 (Li et al., 2010), all of them known to respond to drought and/or salinity stresses. The second group also comprises WRKY proteins implicated in osmotic stresses tolerance (OsWRKY100, NcWRKY53, OsWRKY45) and/or pathogens response (AtWRKY70, BnWRK70, TaWRKY45, OsWRKY45, AtWRKY62, and AtWRKY38) (Kim et al., 2008) (**Figure 1B**), which are differently modulated by SA, jasmonic acid (JA), ethylene and abscisic acid (ABA).

**FIGURE 2:**
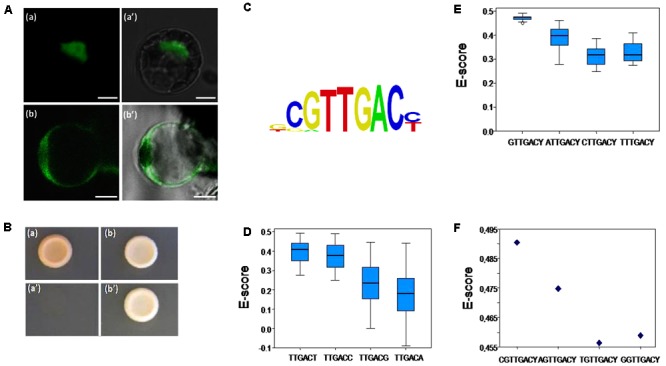
SlWRKY3 subcellular localization, *trans*-regulation properties and DNA-binding specificities. **(A)** Transient expression of YFP-SlWRKY3 fusion protein and control YFP in tomato leaf protoplasts. (a,a′) YFP-SlWRKY3 fluorescence and bright field/YFP fluorescence, respectively. (b,b′) Control YFP fluorescence and bright field/YFP fluorescence, respectively. Scale bar indicates 5 μM. **(B)** SlWRKY3 transactivation ability. *SlWRKY3* coding region was fused to GAL4-DBD into the pGBKT7 vector carrying the nutritional marker *TRP1* as reporter gene. The resulting plasmid, and the empty pGBKT7 vector used as negative control, were transformed into the yeast strain Y8930 harboring *ADE2* and *HIS3* reporter genes. Yeasts were separately grown on SD (synthetic dropout) medium lacking either tryptophan (a,b; pGBKT7 or GAL4-DBD-SlWRKY3, respectively), or adenine and histidine (a′,b′; pGBKT7 or GAL4-DBD-SlWRKY3, respectively). **(C)** Position weight matrix (PWM) representation of the top scoring 8-mer obtained in “seed-and-wobble” algorithm. **(D)** Box plot of the distribution of enrichment scores (*E*-scores) of all possible 8-mers containing the indicated elements. Boxes represent quartiles 25–75%, black line represents the median of the distribution (quartile 50%) and bars indicate quartiles 1 to 25% (above) and 75 to 100% (below). The higher and sharper distributions of *E*-scores corresponding to TTGACT and TTGACC motifs reflect higher binding affinity of SlWRKY3 to these elements. **(E)** Box plot of the distribution of *E*-scores of all possible 8-mers containing the indicated elements varying at their 5′-end nucleotide, showing highest affinity of SlWRKY3 for the DNA element GTTGACY. Representation of the boxes is as in **(D)**. **(F)** *E*-scores of the 8-mers indicated in the Figure. Each point represents the average of two different *E*-scores, corresponding to NGTTGACC and NGTTGACT. Highest affinity was observed for CGTTGACY, as represented in the logo representation in **(C)**. In spite of this, all the 8-mers containing the element GTTGACY were very efficiently recognized by SlWRKY3.

Main *trans*-regulation properties of SlWRKY3 were examined, including subcellular localization, *trans*-activation ability, and DNA-binding specificity. *SlWRKY3* ORF fused in frame in its N-terminal end to the yellow fluorescent protein (YFP) coding sequence under control of the cauliflower mosaic virus *35S* promoter was transiently introduced into tomato protoplasts. As shown in **Figure 2A** (a,a′), the YFP-SlWRKY3 fusion protein was strictly localized in the nucleus, while free YFP was distributed throughout the cytosol in addition to the nucleus [**Figure 2A** (b,b′)]. These results are in agreement with the expected role of TFs.

Next, SlWRKY3 capacity to activate a downstream gene expression was assessed in yeast. *SlWRKY3* coding sequence was cloned in frame to the GALACTOSE 4 (GAL4) DNA binding domain (Trp^+^ plasmid) and the construct introduced into the Y8930 yeast strain carrying the *ADENINE 2* and the *HISTIDINE 3* nutritional markers for adenine and histidine auxotrophy. As observed in **Figure 2B**, yeasts transformed with *SlWRKY3* were able to rapidly grow on control medium lacking tryptophan [**Figure 2B** (b)] and on selective medium lacking histidine and adenine [**Figure 2B** (b′)]. In contrast, yeasts carrying an empty vector could only grow on medium devoid of tryptophan [**Figure 2B** (a,a′)]. These results indicate that SlWRKY3 is a strong transcriptional activator.

Finally, SlWRKY3 DNA binding specificities were assessed *in vitro* according to a protein binding microarray approach (Godoy et al., 2011). SlWRKY3 coding sequence was fused in frame to the maltose-binding protein, and the fusion protein hybridized to the Protein-Binding-Microarray 11 (Godoy et al., 2011). Results indicate that SlWRKY3 binds to the consensus CGTTGACY (Y, pyrimidine) element (**Figure 2C**) that contains the core W-box described for other WRKY TFs (Ciolkowski et al., 2008). Likewise, the binding preferences to C or T at the 3′-end of the W-box were assessed. We observed similar binding affinity for W-boxes containing any of the pyrimidine residues, although a slight preference for T was observed (**Figure 2D**). By contrast, “mutant” W-boxes containing a purine residue at 3′ were poorly recognized by SlWRKY3 (**Figure 2D**). Similarly, a detailed analysis of binding preferences to the W-box with different flanking nucleotides at the 5′-end was conducted. In this case, SlWRKY3 binds more efficiently to W-box containing G at the 5′-end (**Figure 2E**), and even higher to DNA elements with CG at their 5′-end (**Figure 2F**).

**FIGURE 3:**
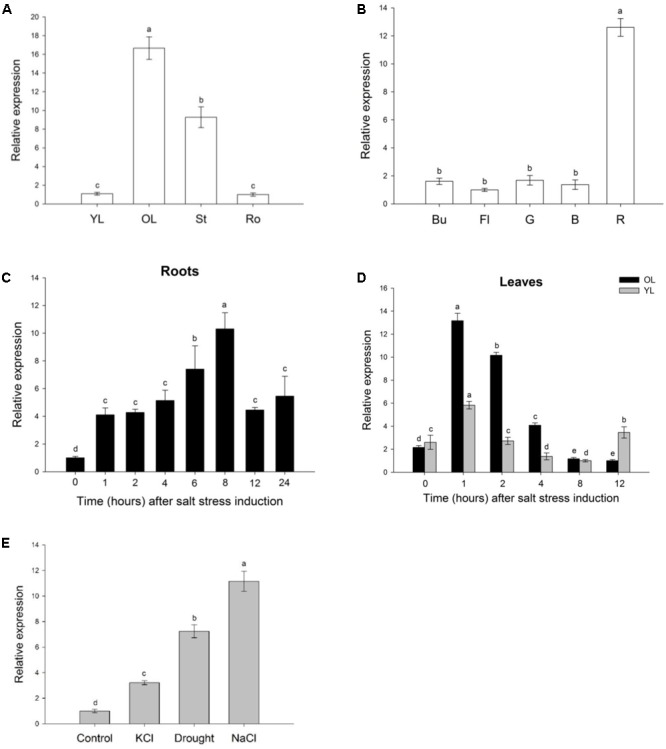
Analysis of *SlWRKY3* expression profile. QRT-PCR analysis of *SlWRKY3* spatio-temporal expression profile during tomato plant development, in vegetative organs **(A)** and reproductive organs **(B)**. QRT-PCR analysis of *SlWRKY3* expression pattern in response to salinity (150 mM NaCl) in **(C)** roots or **(D)** leaves (young leaves (YL) and old leaves (OL), separately). *SlWRKY3* weakest relative expression (value is 1) occurs at 8 h for YL and 12 h for OL. **(E)** Expression of *SlWRKY3* in tomato roots exposed to 150 mM NaCl, 150 mM KCl or drought. *Actin* and *GAPDH* were used as internal controls. Data represent means and SDs of three technical replicates (*n* = 4 plants). Different letters indicate significant differences between treatments according to the Student-Newman-Keuls test at *P* < 0.05. Ro, roots; St, stem; Bu, bud; Fl, flower; G, green stage; B, breaker stage; R, red stage of tomato fruit development.

*SlWRKY3* expression profile during plant development was assessed by quantitative RT-PCR (qRT-PCR). In vegetative organs (**Figure 3A**), *SlWRKY3* transcripts accumulate mostly in old leaves (∼16 times more transcripts than in young leaves or roots), followed by the stem. In reproductive organs (**Figure 3B**), *SlWRKY3* is highly expressed in the red fruit, with 12.6 more transcripts than in the flower or during early stages of fruit development (green and breaker stages). Taken together, these results indicate that *SlWRKY3* is predominantly expressed in old leaves and red mature fruit, and could thus be involved in regulation of senescence-related processes.

### *SlWRKY3* Is Induced by Different Osmotic Stresses

To investigate the putative involvement of SlWRKY3 in salt stress response, *SlWRKY3* expression under short-term salinity stress (150 mM NaCl, 24 h) was analyzed by qRT-PCR. In roots, *SlWRKY3* expression was rapidly induced, and gradually increased with time, before peaking 8 h after stress onset with transcripts accumulating 10 times more than in control roots. *SlWRKY3* expression subsequently decreased, but corresponding transcripts remained nearly five times higher 24 h after salt stress beginning than in untreated (T0) roots (**Figure 3C**). In tomato leaves, *SlWRKY3* transcripts accumulation followed a similar pattern in young and old leaves (**Figure 3D**). Indeed, *SlWRKY3* transcripts rapidly accumulated, with a peak of abundance occurring as soon as 1 h after salt stress onset. *SlWRKY3* expression steadily decreased afterward. Twelve hours after stress commencement, *SlWRKY3* transcripts remained slightly similar in salt treated young leaves, and were two times less abundant in old leaves, than in untreated leaves (**Figure 3D**).

*SlWRKY3* expression under additional osmotic stresses was investigated in tomato roots exposed for 3 h to 150 mM NaCl, 150 mM KCl or drought (air drying). As observed in **Figure 3E**, *SlWRKY3* transcripts were the most abundant in case of NaCl treatment (11.15 more transcripts than in untreated roots), followed by drought and KCl treatment. Altogether, these results suggest that *SlWRKY3* is up-regulated by different osmotic stresses.

### SlWRKY3 Is Involved in Salicylic Acid Signaling

**FIGURE 4:**
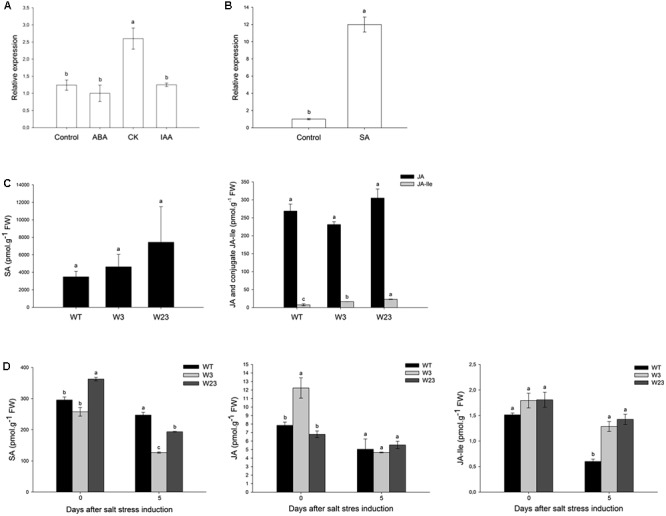
*SlWRKY3* hormonal regulation. **(A,B)** QRT-PCR analysis of *SlWRKY3* expression in response to different hormonal treatments. *EF1*α and *GAPDH* were used as internal controls for ABA, CK, and IAA treatments, while actin and *GAPDH* were used as internal controls for SA treatment. Data represent means and SDs of three technical replicates (*n* = 6 plants). **(C)** Hormonal contents of *35S*::*SlWRKY3* (W3 and W23) and WT tomato seedlings grown *in vitro* on regular MS medium. **(D)** Hormonal contents in Leaf 4 of transgenic and WT plants grown in hydroponics under control conditions or 5 days on 125 mM NaCl. Data represent means and SDs of two readings of the same extract (*n* = 4 plants). Different letters indicate significant differences between treatments or tomato lines according to Student–Newman–Keuls test at *P* < 0.05. SA, salicylic acid; JA, jasmonic acid; JA-Ile, jasmonoyl-isoleucine; CK, cytokinins; ABA, abscisic acid; IAA, indole-3-acetic acid; CK, cytokinin. FW, fresh weight.

Phytohormones including ABA, zeatin (cytokinin, CK), indole acetic acid (auxin, IAA) and SA were separately added to tomato hydroponic cultures. Hormonal effects on *SlWRKY3* expression were analyzed by qRT-PCR on tomato roots 3 h after treatments onset, and compared to untreated plants for ABA, CK, and IAA (**Figure 4A**) or mock plants (treated with 0.044% ethanol) for SA (**Figure 4B**). As reported in **Figure 4A**, CK treatment significantly (*P* < 0.05) enhanced 2.5 times *SlWRKY3* transcripts accumulation compared to untreated roots. Similarly, after SA treatment, *SlWRKY3* transcripts increased 12 times compared to mock plants. Neither exogenous ABA nor IAA hormones could remarkably modify *SlWRKY3* expression in the tested conditions.

To gain further insights onto *SlWRKY3* hormonal regulation, tomato overexpressing *SlWRKY3* ORF driven by the CaMV*35S* promoter were generated (described below). Hormonal contents of 3-weeks old seedlings (four leaves stage) grown *in vitro* were analyzed in two F2 transgenic lines (W3 and W23) and compared to wild-type (WT) plants (**Figure 4C**). Under normal conditions of growth, *SlWRKY3*-transgenic tomatoes accumulated a higher mean SA contents (4624 pmol/g FW and 7429 pmol/g FW for W3 and W23, respectively) than WT (3888 pmol/g FW). Over-accumulation of SA is often correlated with reduced amounts of JA. Comparable JA contents were, however, measured in transgenic tomatoes (231 pmol/g FW and 305 pmol/g FW for W3 and W23, respectively) and WT (268 pmol/g FW) (**Figure 4C**). In contrast, the JA conjugate jasmonoyl-isoleucine (JA-Ile), representing the bioactive form of the hormone, was more profuse (*P* < 0.05) in transgenic tomatoes than in WT.

Total phytohormones were then extracted from Leaf 4 of plants grown in hydroponics under control conditions (no treatment) or 5 days after stress (125 mM NaCl) initiation (**Figure 4D**). In the absence of salt treatment, W23 and W3, respectively, accumulated more and less SA than WT. Salinity decreased SA contents for all type of plants and after 5 days, W3 and W23 accumulated up to 50% less SA than WT. Similar results have been described for JA, since JA contents were higher in W3 (12 pmol/g FW) than W23 or WT under control conditions, and salt negatively affected JA accumulation for WT, W3 and W23. Finally, before stress application, all lines accumulated comparable JA-Ile contents (∼1.5 pmol/g FW). Although salt stress decreased JA-Ile contents in WT and transgenic plants, these concentrations remained up to 60% higher in W3 and W23 than in WT.

### *35S*::*SlWRKY3* Tomatoes Show Improved Tolerance to Salinity

In Arabidopsis, *SlWRKY3* overexpression improved plant germination efficiency in presence of NaCl, mannitol and KCl (Supplementary Figure S1). Functional analysis of SlWRKY3 was subsequently assessed in homologous system. *SlWRKY3* was introduced in tomato under control of the CaMV*35S* promoter as reported above. Analyses were conducted on the three F2 transgenic lines W3, W9, and W23, and compared to WT. Tolerance of transgenic and WT tomatoes to long term salinity constraint (125 mM NaCl, 20 days) was investigated using hydroponic cultures. All type of plants were in parallel cultivated in control conditions (no treatment) (Supplementary Figures S2–S4).

**FIGURE 5:**
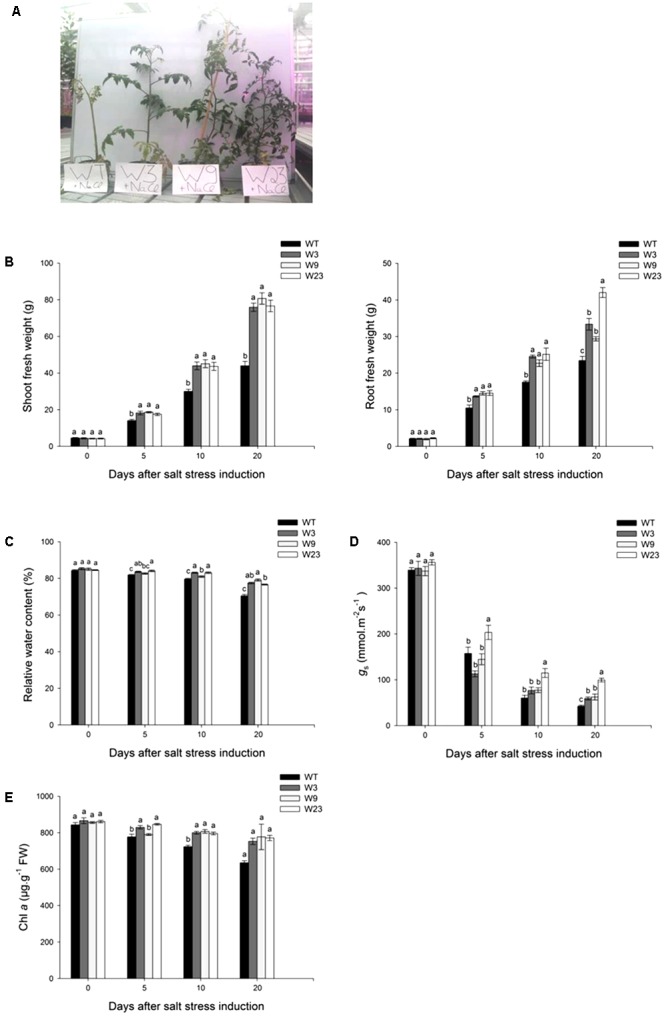
Evaluation of growth and photosynthesis parameters of *35S*::*SlWRKY3* (W3, W9, and W23) and WT tomato plants exposed to long-term salt stress (125 mM NaCl, 20 days). **(A)** Phenotype of *35S::SlWRKY3* and WT plants exposed to 20 days salinity. **(B)** Shoot and root fresh weight. **(C)** Relative water content and **(D)** Leaf stomatal conductance (*g*_s_) of Leaf 4. **(E)** Chlorophyll *a* (Chl *a*) contents of Leaf 5. Data represent means ± SEs of four biological replicates. Letters indicate values that significantly differ between *SlWRKY3* transgenic tomatoes and WT according to Student–Newman–Keuls test at *P* < 0.05.

Compared to control conditions (Supplementary Figure S2), salinity affected shoot and root growth (**Figures 5A,B**). Before stress initiation, transgenic and WT lines presented comparable aerial part (∼4 g) and roots (∼2 g) weight. As soon as 5 days after salt stress beginning, W3, W9, and W23 plants grew significantly (*P* < 0.05) better than WT. Twenty days after stress initiation, the weight of WT plants shoot was up to 45% less than that of transgenic lines (**Figure 5B**). A similar tendency was observed for roots, since transgenic plants significantly (*P* < 0.05) displayed during salinization term a higher formation of roots fresh weight (FW) than WT. All transgenic lines significantly (*P* < 0.05) showed in addition during stress period higher relative water content of Leaf 4 than WT (**Figure 5C**). Furthermore, leaf 4 stomatal conductance of transgenic and WT tomatoes was similar (∼350 mmol/m^2^.s) before salt stress onset (**Figure 5D**). Five days after stress establishment, *g*_s_ started to decrease by around 50% for all type of lines, and even to less than 20% of its initial value 10 days after stress beginning. Finally, after 20 days of exposure to salinity, W3 (59.3 mmol/m^2^.s), W9 (62.5 mmol/m^2^.s), and W23 (99.5 mmol/m^2^.s) presented a better *g*_s_ than WT (42.3 mmol/m^2^.s). Evaluation of chlorophyll *a* (Chl *a*) contents of Leaf 4 were also in agreement with positive effects of *SlWRKY3* overexpression on photosynthesis efficiency. Salinity reduced indeed Chl *a* contents in all tomato lines (**Figure 5E**). However, these contents remained significantly (*P* < 0.05) higher in *35S::SlWRKY3* than in WT.

**FIGURE 6:**
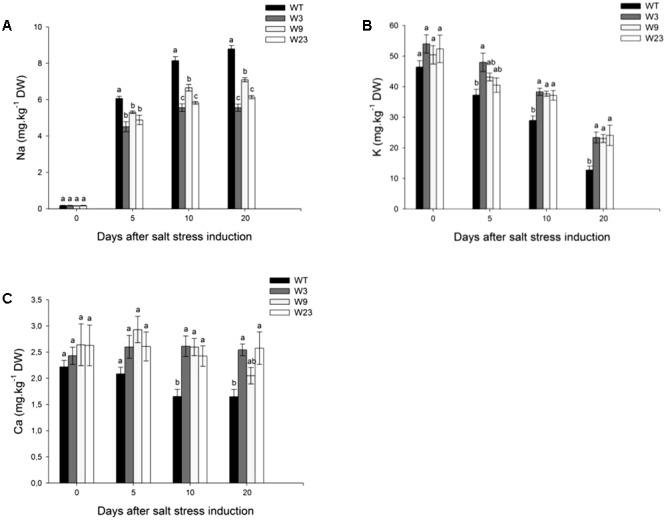
Ions accumulation in Leaf 4 of *35S*::*SlWRKY3* (W3, W9, and W23) and WT tomato plants exposed to long-term salt stress (125 mM NaCl, 20 days). **(A)** Sodium (Na), **(B)** Potassium (K), and **(C)** Calcium (Ca) contents. Data represent means ± SEs of four biological replicates. Letters indicate values that significantly differ between *SlWRKY3* transgenic tomatoes and WT according to Student–Newman–Keuls test at *P* < 0.05.

One strategy of glycophyte plants to cope with the ionic component of salt stress comprises selective exclusion of Na^+^, and maintenance of high K^+^ or Ca^2+^ concentrations necessary to proper cellular metabolism and enzymatic activity. Before stress initiation, WT and transgenic tomatoes accumulated less than 0.2 mg/kg dry weight (DW) Na^+^ (**Figure 6A**). Sodium contents increased in all type of plants over stress period, and 5 days after stress onset, transgenic lines significantly (*P* < 0.05) exhibited lower contents of Na^+^ as compared to WT. Comparable tendencies were observed 10 and 20 days after treatment onset. A similar analysis of K^+^ and Ca^2+^ content was conducted on the same leaf 4. Before salt application, all type of lines showed similar K^+^ contents (between 46 and 54 mg/kg DW) (**Figure 6B**). These concentrations decreased with time and 20 days after salinity onset, WT exhibited the lowest concentration of K^+^. Salinity affected as well calcium accumulation to a much greater extent in WT than in transgenic lines (**Figure 6C**). Altogether, these results showed a limited Na^+^ accumulation in *SlWRKY3* gain-of-function tomatoes over salinity stress period, together with preservation of K^+^ and Ca^2+^ balance.

**FIGURE 7:**
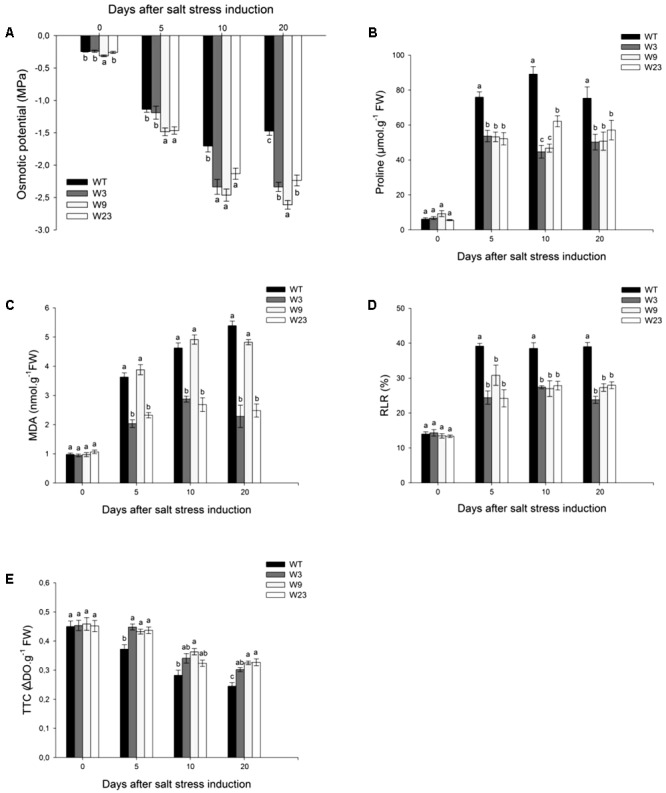
Evaluation of oxidative-stress related parameters of *35S*::*SlWRKY3* (W3, W9 and W23) and WT tomato plants exposed to long-term salt stress (125 mM NaCl, 20 days). **(A)** Osmotic potential of Leaf 5. **(B)** Proline and **(C)** Malonyldialdehyde (MDA) contents of Leaf 5. **(D)** Relative leakage rate (RLR) and **(E)** 2,3,5-triphenyltetrazolium chloride (TTC) staining measured on Leaf 5. Data represent means ± SEs of four biological replicates. Letters indicate values that significantly differ between *SlWRKY3* transgenic tomatoes and WT according to Student–Newman–Keuls test at *P* < 0.05.

In the absence of stress, Leaf 5 osmotic potential Ψs was ranging between -0.23 and -0.31 MPa for WT and transgenic lines. Ψs values remained statistically lower at 10 and 20 days after stress initiation for all transgenic lines than for WT (**Figure 7A**). This prompted us to examine which metabolites could differentially accumulate between WT and transgenic lines. Before salt treatment, WT and transgenic lines accumulated less than 10 μmol/g FW proline (**Figure 7B**). Salinity dramatically increased proline contents for all type of tomato plants as early as 5 days post-stress, but up to 50% more for WT than for transgenic lines.

Next, effects of induced senescence due to sodium toxicity were assessed, including membrane lipid peroxidation which was evaluated through malonyldialdehyde (MDA) quantification (**Figure 7C**). Before stress onset, WT and transgenic plants showed similar MDA values (∼1 nmol/g FW). Salinity strongly increased MDA contents in all plants, and 20 days after salt stress onset, W3 (2.3 nmol/g FW) and W23 (2.5 nmol/g FW) showed up more than 50% less MDA than WT (5.4 nmol/g FW) or W9 (4.8 nmol/g FW). Likewise, evaluation of the relative leakage rate (RLR) revealed that *35S::SlWRKY3* lines displayed over stress period a lower RLR than WT (**Figure 7D**). Reduction of 2,3,5-triphenyltetrazolium chloride (TTC) to formazan is also a direct index of plant tissue viability. No difference was recorded between transgenic lines and WT in the absence of stress (**Figure 7E**). Salinity induced a progressive decrease in TTC reduction for all types of lines. However, tissue viability remained over stress period significantly higher for transgenic than for WT tomatoes (**Figure 7E**).

In summary, upon salinization, *35S::SlWRKY3* transgenic plants display a better osmotic adjustment, detoxification of reactive oxygen species (ROS), and membrane protection than WT.

### Transcriptome Analysis of Tomato *SlWRKY3*-Overexpressing Lines

**FIGURE 8:**
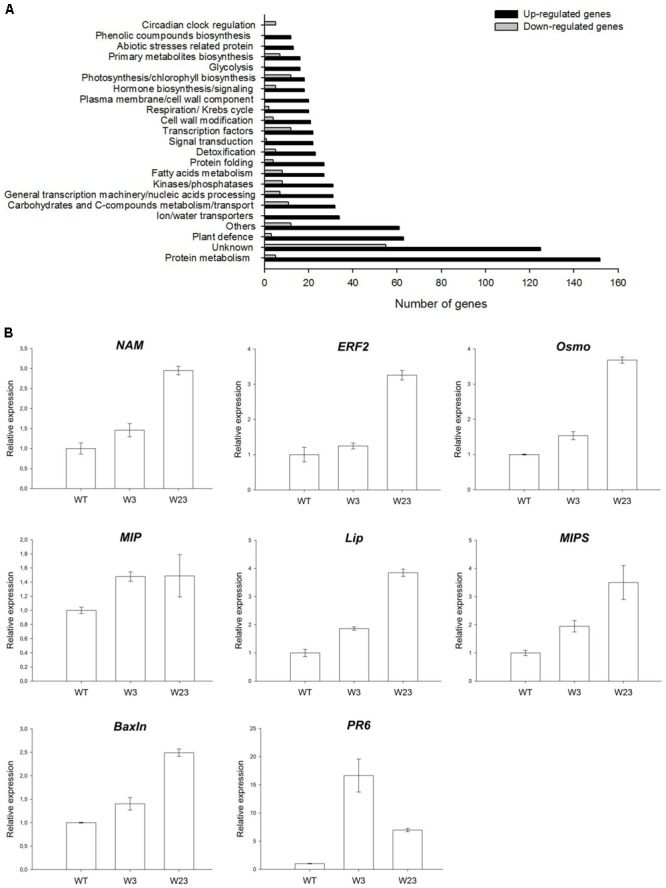
Microarray analysis of differentially regulated genes between WT and W3-line tomatoes. **(A)** Number and categories of genes up- and down-regulated in tomato W3 transgenic line compared to WT. **(B)** QRT-PCR analysis of SlWRKY3 putative target genes in transgenic tomato lines W3 and W23, comparatively to WT. *Actin* and *GAPDH* were used as internal controls. Data represent means and SDs of three technical replicates (*n* = 4 plants). NAM (No Apical Meristem) transcription factor; ERF2, Ethylene Response Factor 2; Osmo, osmotin; MIP, membrane intrinsic protein (aquaporin); Lip, lipase; MIPS, myo-inositol phosphate synthase; BaxIn, BAX Inhibitor; PR6, pathogenesis-related 6.

A commercial Affymetrix tomato microarray representing 43,803 tomato probes was used to find out differentially expressed genes between WT and the W3 transgenic line. Analysis was conducted on tomato seedlings grown *in vitro* under normal growth conditions and allowed identification of 804 up-regulated and 166 down-regulated genes differentially (*P* ≤ 0.05) expressed between W3 and WT (**Figure 8A**).

Among differentially up-regulated genes, 19 were encoding TFs, with 5 of them belonging to the large AP2 family, and 3 to the bZIP-type regulators (**Figure 8A** and **Table 1**). Additional families of TFs were also represented such as zinc-finger, NAM/NAC, and WRKY. Similarly, down-regulated TFs included zinc-finger, WRKY, basic Helix-Loop-Helix (bHLH) and MADS-box members, among others, indicating that constitutive expression of *SlWRKY3* affected different types of downstream regulators (**Table 1**). Genes related to osmotic stress responses were of particular interest. Around 25 genes related to abiotic stresses response were differentially up-regulated in the W3 line, and included dehydration, wound, cold and salt stress responses proteins, as well as chaperones (**Table 2**). Interestingly, 34 genes coding for aquaporins and ions transporters were also significantly induced in W3 compared to WT, while detoxification enzymes such as glutathione-*S*-transferases and peroxidases were represented by 23 genes (**Table 2**).

**Table 1:** Transcription factors encoding genes differently up-regulated or down-regulated (*P* ≤ 0.05) between *SlWRKY3*-transgenic tomato line W3 and WT.

Accession	Transcription factor family	Log2 fold
**Up-regulated genes**	
AK322452	DREB3	2.5
BP893968	AP2d	2.7
AK320001	RAV2	2.7
AK319979	Pti4	3.4
AW034241	ERF2	4.3
AW651406	bZIP	2.6
AK224603	bZIP	2.8
AK326827	bZIP	3.1
EU636698	CONSTANS-like Zinc Finger	3.5
AJ784616	Stress-associated protein 6 (Zinc finger)	6.7
BG627283	BTF3-like (NAC)	2.9
AK324393	NAM-like	5
BM412776	WRKY	5.8
AK322575	WRKY3	14.5
TC239950	TCP	2.5
AK319300	TCP transcription factor 14	3
TA41176_4081	GRAS 6	3.3
AK325371	Putative transcription factor	3.4
BP879218	Putative Squamosa promoter-binding protein	3.5
**Down-regulated genes**	
DB690655	CONSTANS-LIKE 16 Zinc Finger	3.9
DB697244	CONSTANS-LIKE 9 Zinc Finger	5.5
AW624869	CONSTANS-LIKE 10 Zinc Finger	6.8
AW622759	WRKY	3.9
AK247980	WRKY	5.2
AK247135	PIF1 (bHLH)	2.5
BI935893	putative bHLH	2.4
AK327992	MADS box	4
AK327530	PHD finger	2.2
BI925618	Bell-like homeodomain protein 2 (POX domain)	2.2
BI921917	Tubby-like F-box protein	2.6

**Table 2:** Up-regulated genes differently (*P* ≤ 0.05) expressed between *SlWRKY3*-line W3 and WT, and related to osmotic stress tolerance and plant defense.

Accession	Description	Log2 fold
**Stress-related proteins**
AK329158	Putative dehydration-responsive protein RD22	10.4
TA36603_4081	Chaperone Hsp90-1	10.1
AK329506	Wound/stress induced protein	8.3
BG734684	Heat shock protein 70	5.4
TA41419_4081	Low temperature and salt responsive protein	4.3
AK328356	Universal stress protein A-like protein	4
TA38285_4081	Caffeic acid *O*-methyltransferase II	10.5
AK323694	Cinnamyl alcohol dehydrogenase	5
AW035414	Phenylalanine ammonia lyase	2.6
**Water/ions transporters**
AK324945	Inorganic phosphate transporter	7.4
AK322565	Metal transporter	6.1
AK321157	Putative high-affinity nitrate transporter	5.3
AW093618	Vacuolar cation/proton exchanger 3-like	4.9
AK322020	Ca^2+^/H^+^-exchanging protein	4.8
BG130774	PIP-type aquaporin	4
NP000693	Sodium/potassium-transporting ATPase	2.9
AK319783	Vacuolar H^+^-ATPase	3
**Detoxification**
AK325343	Glutathione *S*-transferase	8
AK323682	Glutathione *S*-transferase	7.9
BT013000	Peroxidase	7.8
TA38392_4081	Peroxidase	7.3
AK327211	Glutathione *S*-transferase	5.6
**Hormones biosynthesis/signaling**
TA36689_4081	ACC oxidase	10.6
AW217769	Salicylic acid carboxyl methyltransferase	6.4
AK324766	Putative gibberellin 20 oxidase 1-like	5.8
AK224709	SAUR family protein	4.3
AK319805	LAX3	4.2
AK323740	ACC synthase 2	3
AK224691	Jasmonate ZIM-domain protein 3	2.8
**Fatty acids metabolism**
AW622151	Lipase/esterase	21.2
AK329281	Putative acyl-CoA synthetase	6.5
AK319365	Monogalactosyldiacylglycerol synthase 2	5.6
GO375192	Stearoyl-ACP desaturase	4.8
AK319952	Delta-9 fatty acid desaturase	4.5
**Plant defense**
AK322366	Osmotin (PR P23)	16.2
AW224719	NRC1/NBS-LRR type resistance protein	18.3
GO373278	Endochitinase	8.2
DB694032	Defensin	7.3
Y08804	Pathogenesis Related 6 (PR6)	6.6
AW031662	Glucan endo-1,3-beta-glucosidase B	6.6
AK247569	DCD (Development and cell Death) regulator	5.6
AK322997	Bax inhibitor	3
**Cell wall modification**
AJ560647	Expansin	5.9
BP904709	Pectin methylesterase	5
AK320289	GDP-mannose pyrophosphorylase	4.2
AK321776	Beta-glucosidase 01	3.1
AY497475	Xyloglucan endotransglucosylase-hydrolase XTH5	3
**Kinases**
BP908967	Serine/threonine-protein kinase	7.1
AI773030	LRR receptor-like serine/threonine-protein kinase	5.2
AK329792	Mitogen-activated protein kinase 3	4.3
AK325830	Serine/threonine-protein kinase	4.2
AW220068	CBL-interacting serine/threonine-protein kinase	3.3
AK319220	CBL-interacting serine/threonine-protein kinase	2.6

Differentially expressed genes related to hormone biosynthesis/signaling were likewise picked up (**Table 2**). This group encompassed genes coding for ACC oxidase and ACC synthase involved in ethylene biosynthesis, salicylic acid carboxyl methyltransferase (which catalyzes the formation of SA methyl salicylate involved in plant defense response), auxin-responsive SAUR (small auxin up RNA) and LAX3 (auxin influx carrier) proteins, as well as the jasmonate ZIM-domain protein 3, a repressor of the jasmonate pathway.

Additionally, it is noteworthy that 63 up-regulated genes encoded proteins related to plant defense (**Table 2**), and included Pathogenesis-Related (PR) proteins, regulator of development and cell death (DCD) processes, in addition to enzymes involved in degradation of pathogens cell wall. Around 35 genes encoding proteins involved in lipid metabolism were moreover significantly up-regulated in the W3 line compared to WT. Finally, more than 30 genes encoding proteins involved in phosphorylation (kinases, and to a lesser extent phosphatases) differentially showed more transcripts in the W3 line.

To validate the microarray data, eight genes were selected for further qRT-PCR analysis and accumulation of the corresponding transcripts measured in seedlings of WT and compared to lines W3 and W23 (**Figure 8B**). These genes encoded NAM (AK324393) and ERF2 (AW034241) TFs, lipase (AW622151), aquaporin (BG130774), myo-inositol phosphate synthase (MIPS, AK321761), PR6 (Y08804), osmotin (AK322366), and Bax inhibitor (AK322997). In accordance with microarray data, all tested genes were up-regulated in W3 and W23 compared to WT (**Figure 8B**).

We next evaluated the presence of SlWRKY3-binding elements in the promoters of genes up-regulated in the W3 line. We selected up-regulated genes presented in **Tables 1, 2** and recovered their promoter sequences (1 and 3 kb) from available tomato databases. The core W-box (TTGACY) was slightly more represented in the promoters of genes up-regulated than the corresponding “mutant” element (TTGACR), considering 1 and 3 kb promoter regions. This tendency was more pronounced in the case of extended motifs containing G and CG residues at their 5′-ends (**Table 3**).

**Table 3:** Presence of W-boxes in the promoters of genes up-regulated in *SlWRKY3*-overexpressing W3 line.

	1 kb		3 kb	
	Number of elements	% Promoters with elements	Number of elements	% Promoters with elements
TTGACY	85	72.7	236	95.4
TTGACR	58	62.1	161	90.9
GTTGACY	17	24.2	38	43.9
GTTGACR	4	6.1	20	27.3
CGTTGACY	4	6.1		7.6
CGTTGACR	0	0	5 0	0

## Discussion

Although WRKY involvement in abiotic stress tolerance has been established (Chen et al., 2012; Liu et al., 2013; Ding et al., 2014; Qin et al., 2015), a WRKY complete characterization has never been conducted so far in tomato, and has never involved physiological, biochemical and transcriptomics data together (Supplementary Figure S5). *SlWRKY3* is rapidly induced in roots and leaves by salt treatment, as well as by drought and KCl. SlWRKY3 is assigned to tomato Group III, where 4 out of the 11 members are strongly induced by salinity (Huang et al., 2012). SlWRKY3 may or may not directly regulate all the panoply of stress-related genes necessary to salinity response (**Table 3**), as it is a strong transcriptional activator (**Figure 2B**). In Arabidopsis, mutation of *AtWRKY8* resulted in plants hypersensitivity to salt, and decreased expression of stress-related genes such as *RD29A* or *RD29B* (Hu et al., 2013). AtWRKY8 binds to *RD29A* promoter under salt stress conditions (Hu et al., 2013), suggesting that WRKYs directly interfere with stress-related genes expression. Likewise, soybean GmWRKY27 improves plant tolerance to salinity via modulation of the expression of several stress-related genes, and directly binds to the promoter of *GmNAC29*, a negative effector of stress tolerance (Wang F. et al., 2015). In addition, several types of TFs more or less known to participate in osmotic stresses tolerance in tomato are differentially regulated in *SlWRKY3*-transgenic tomatoes compared to WT. AP2/ERF regulators such as SlDREB2 (Hichri et al., 2016), bZIP (Orellana et al., 2010), zinc-fingers (Hichri et al., 2014), and NAC (Zhu et al., 2014) are important actors of salinity tolerance in tomato by modulation of stress-related genes expression.

Salinity impacts multiple aspects of plant growth and development by imposing an osmotic stress followed by toxicity effects due to sodium and chloride ions. Diverse cellular mechanisms are consequently deployed in plants and include biosynthesis of compatible solutes such as proline necessary to maintain cell turgor and preserve enzymatic activities/cellular structures (Parida and Das, 2005). In *SlWRKY3* transgenic tomatoes, proline contents were similar to that of WT before stress, increased following exposure to salinity, but remained lower than in WT. Similar results were reported following *SlDREB2* overexpression in tomato, where salinity tolerance was associated in transgenic plants with reduced proline contents over stress period compared to WT (Hichri et al., 2016). However, salinity tolerance is associated in tobacco overexpressing *TaWRKY44* with increased proline contents (Wang X. et al., 2015), revealing that plants differently face osmotic stress adaptation. Since *SlWRKY3*-transgenic tomatoes displayed during stress period smaller osmotic potential than WT, they may thus accumulate other secondary metabolites interfering with osmotic stress management, such as sugars for instance (Wang X. et al., 2015; Agarwal et al., 2016).

Among additional processes required for salt tolerance, protection of cell membrane from oxidation, in addition to scavenging of ROS (superoxide and hydroxyl radicals, hydrogen peroxide), are of particular importance. *SlWRKY3* overexpressors were able to better withstand oxidative stress than WT, by maintaining lower contents of MDA and RLR percentage, together with higher TTC. High basal expression, in absence of any stress, of antioxidant enzymes (GSTs and peroxidases notably) encoding genes in *SlWRKY3* transgenic plants compared to WT as suggested by microarray profiling data (**Table 2**), could possibly explain the reduced membrane lipid peroxidation since no need for *de novo* biosynthesis of these enzymes in case of salinity would be required for the establishment of protective mechanisms. Under salt and drought constraints, *35S::SlWRKY* tobacco plants similarly displayed a lower electrolyte leakage as well as reduced MDA contents compared to WT due higher activities of superoxide dismutase and peroxidase (Li et al., 2012), and similar results have been reported in tobacco overexpressing tomato SpWRKY1 (Li et al., 2015). In general, WRKY TFs control oxidative stress in different plant species (Liu et al., 2013; Wang et al., 2013; Zheng et al., 2013; Ding et al., 2014; Shi et al., 2014; Agarwal et al., 2016).

In W3 transgenic tomatoes, the fatty acids biosynthesis and metabolism pathway were highly induced compared to WT, which may contribute to salinity tolerance as well. Membrane restructuration through augmentation of the unsaturation, and thus maintenance of the membrane fluidity, constitutes indeed a mechanism of adaptation reported for extremely halotolerant yeast-like fungus (Gostincar et al., 2009). In Arabidopsis, the lipase encoding gene *AtLTL1* is induced by osmotic stresses, and its overexpression substantially improved plant germination, growth and reproduction under salinity (Lu et al., 2010). In tomato, overexpression of the reticulum endoplasmic-type omega-3 fatty acid desaturase (LeFAD3) encoding gene improved plant growth and oxidative status in presence of NaCl (Wang et al., 2014).

Glycophyte species do likewise achieve multiple strategies to minimize toxic effects due to sodium accumulation, such as preferential transport and sequestration of Na^+^ into the vacuoles of old leaves, Na^+^ exclusion from roots and stem retention, or maintenance of high K^+^ contents (Volkmar et al., 1998; Parida and Das, 2005; Zhou et al., 2015). Among the mechanisms of Na^+^/K^+^ homeostasis and compartmentalization regulation, the salt overly sensitive (SOS) system is probably the best described (Yang et al., 2009; Núñez-Ramírez et al., 2012). According to our microarray data, a Na^+^/K^+^-transporting ATPase, as well as a vacuolar cation/proton exchanger, a vacuolar H^+^-ATPase, and a Ca^2+^/H^+^-exchanging protein, are up-regulated in W3 tomatoes, and could thus be putatively involved in sodium exclusion and pH homeostasis regulation. In fact, regulation of homeostasis may also be illustrated by potassium and calcium, which accumulates more in all transgenic plants at high salinity than in WT in order to counter the toxic effects of sodium. Critical role of K^+^ in salt stress alleviation has been well documented (Shen et al., 2015), and positive effects of Ca^2+^ on salt stress tolerance have also been reported (Navarro et al., 2000; Nie et al., 2015). Two CBL (calcineurin B-like calcium sensor)-interacting serine/threonine-protein kinases (CIPK) transducing Ca^2+^ signals are additionally induced in W3 transgenic tomatoes. In Arabidopsis, CIPK21 positively regulates salt stress response by controlling water and ion homeostasis across the tonoplast (Pandey et al., 2015). Together, these data strongly support the hypothesis that regulation of sodium, potassium and calcium homeostasis is crucial for salt stress management.

In addition to its involvement in salinity response/tolerance, our results indicate that *SlWRKY3* is also induced in tomato roots following drought stress. Putative involvement of SlWRKY3 in drought response/tolerance can be supported by the elevated number of aquaporin encoding genes up-regulated in *SlWRKY3*-transgenic tomatoes. Three aquaporin encoding genes were highly induced in Arabidopsis plants overexpressing *ThWRKY4*, a positive regulator of salinity tolerance in halophytes (Zheng et al., 2013). Transgenic tobacco plants overexpressing the aquaporin encoding genes *TdPIP1;1* and *TdPIP2* of durum wheat displayed improved tolerance to abiotic and salinity stresses (Zheng et al., 2013), emphasizing the role of plant water status in stress tolerance. In addition, aquaporin overexpression also interferes with plant oxidative status (Chang et al., 2016) and Na^+^/K^+^ homeostasis (Wang L. et al., 2015). In our current study, expression of the *MIP* aquaporin tested (**Figure 8B**) is strongly repressed by salinity (150 mM NaCl, 12 h) in tomato roots. Counteracting the repression of aquaporin encoding genes by induction of its constitutive expression would thus help to rescue salt-stress effects.

## Conclusion

We provided in our study different types of data to support SlWRKY3 involvement in salt stress response in tomato. To gain further insights onto SlWRKY3 mechanisms and better understand its regulatory role, analysis of *slwrky3* mutants, accumulation of NaCl in transgenic plants roots as well as transcriptomic data in presence of salt, are required. A specific focus was given in this study to salinity, but SlWRKY3 involvement in additional environmental and biotic stresses tolerance can further be considered. This latter hypothesis is supported by the study of Huang et al. (2012), demonstrating that *SlWRKY3* (*WRKY81*) is induced by different types of pathogens.

## Author Contributions

IH, YM, and SL designed the research. EZ, PD, and VM performed the hormonal analysis of the plant material and helped in the treatment and interpretation of the data. EG performed the physiological characterization of the selected plant material. JF-Z, IL-V, and RS performed the protein purification and protein binding microarray. AC performed the microarray analysis. AE performed the cellular localization of the YFP–SlWRKY3 fusion protein.

## Conflict of Interest Statement

The authors declare that the research was conducted in the absence of any commercial or financial relationships that could be construed as a potential conflict of interest.
