# Supplementary material for: The Solanum lycopersicum WRKY3 Transcription Factor SlWRKY3 Is Involved in Salt Stress Tolerance in Tomato
Source: Front Plant Sci. 2017 Jul 31;8:1343. doi: 10.3389/fpls.2017.01343 (PMC5534461; doi:10.3389/fpls.2017.01343)
Supplement: Supplementary file 1 [file Data_Sheet_1.doc]

***Supplementary Material***

**The *Solanum lycopersicum* WRKY3 Transcription Factor is Involved in Salt Stress Tolerance In Tomato**

**Imène Hichri, Yordan Muhovski, Eva Žižková, Petre I. Dobrev, Emna Gharbi, Jose Manuel Franco-Zorrilla, Irene Lopez-Vidriero, Roberto Solano, André Clippe, Abdelmounaim Errachid, Vaclav Motyka, Stanley Lutts^*^**

***Corresponding author**:

Pr Stanley Lutts: Earth and Life Institute - Agronomy (ELI-A), Université catholique de Louvain (UCL), B-1348 Louvain-la-Neuve, Belgium. Tel: + 32 10 47 20 37; e-mail: [Stanley.Lutts@uclouvain.be](mailto:Stanley.Lutts@uclouvain.be)


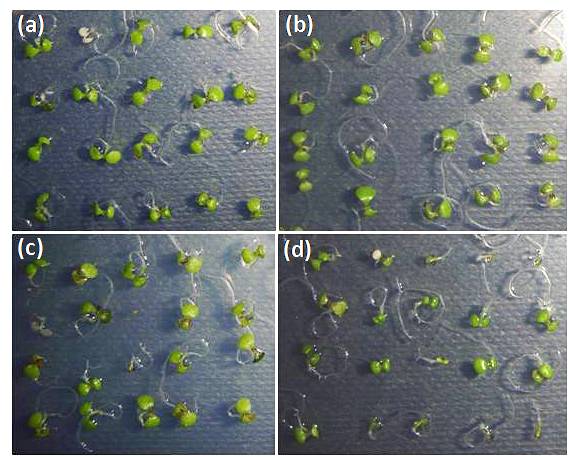

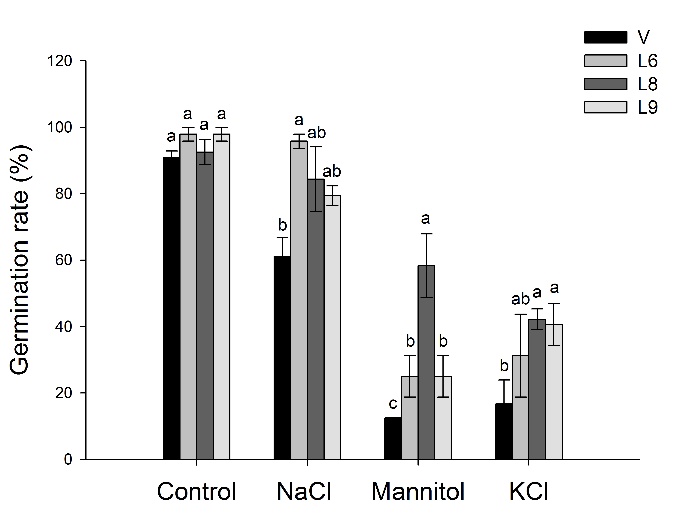


**A**

**B**

**Figure S1 |** *SlWRKY3* positively regulates osmotic stress tolerance in Arabidopsis. A) Germination percentage of V (empty vector) and *35S::SlWRKY3* (L6, L8 and L9) transgenic lines on half-strength MS medium (control) or medium supplemented with 50 mM NaCl, 300 mM mannitol and 150 mM KCl. B) Phenotype five days after sowing of L6 (a), L8 (b), L9 (c) and V (d) on 50 mM NaCl.

Data represent means ± SEs of three biological replicates (n=20 plants). Letters indicate values that significantly differ between Arabidopsis lines according to Student-Newman-Keuls test at p < 0.05.

**A**


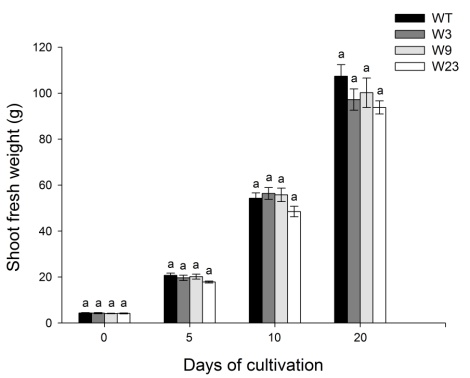

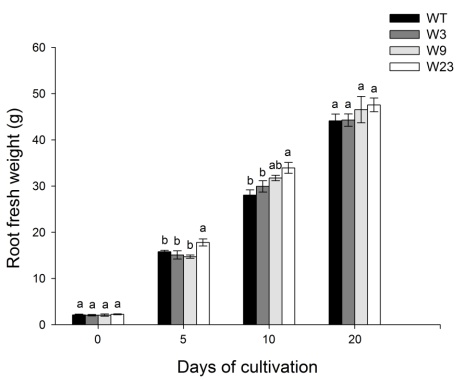

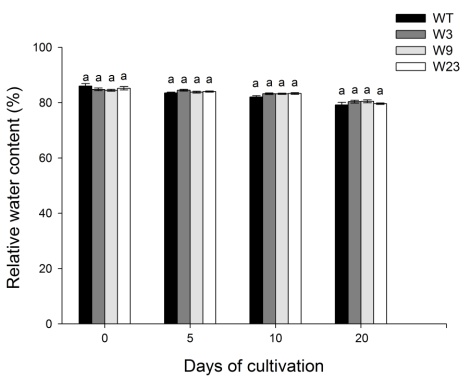

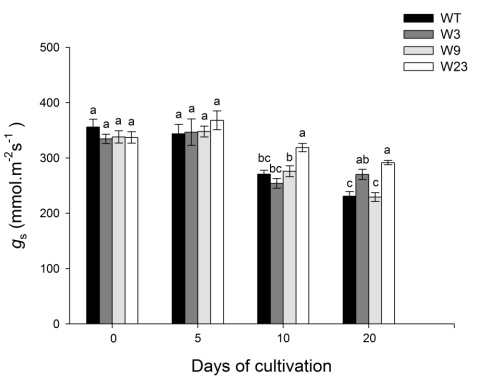

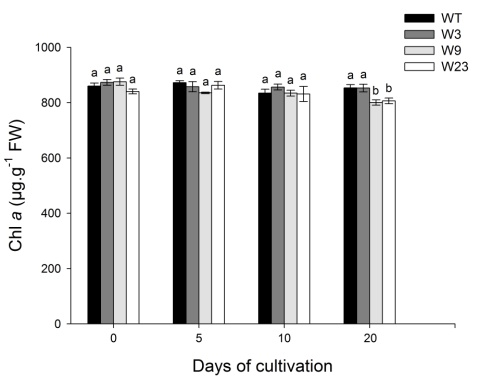


**B**

**C**

**D**

**Figure S2 |** Evaluation of growth and photosynthesis parameters of *35S*::*SlWRKY3* (W3, W9 and W23) and WT tomato plants under control conditions of growth. A) Shoot and root fresh weight. B) Leaf 4 relative water content. C) Leaf 4 stomatal conductance (*g*_s_). (d) Chlorophyll *a* (Chl *a*) contents of Leaf 5.

Data represent means ± SEs of four biological replicates. Letters indicate values that significantly differ between tomato lines according to Student-Newman-Keuls test at p<0.05.


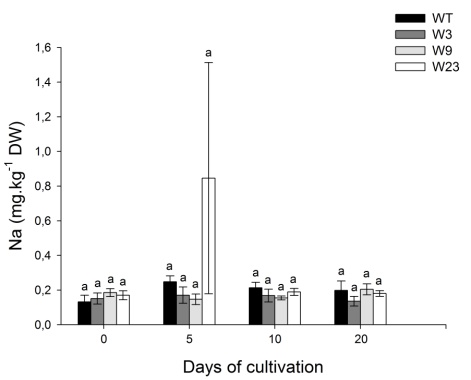

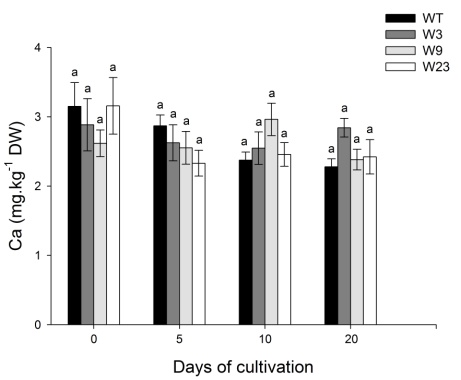

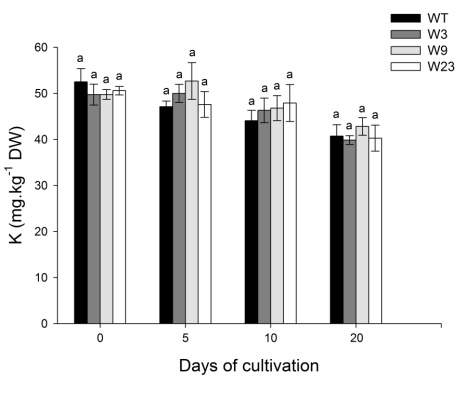


**A**

**B**

**C**

**Figure S3 |** Ions accumulation in Leaf 4 of *35S*::*SlWRKY3* (W3, W9 and W23) and WT tomato plants grown under control conditions. A) Sodium (Na), B) Potassium (K), and C) Calcium (Ca) contents.

Data represent means ± SEs of four biological replicates. Letters indicate values that significantly differ between tomato lines according to Student-Newman-Keuls test at p<0.05.


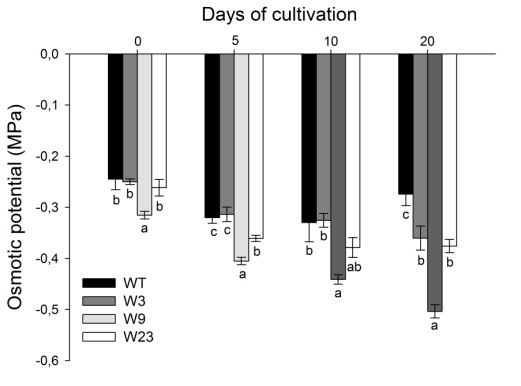

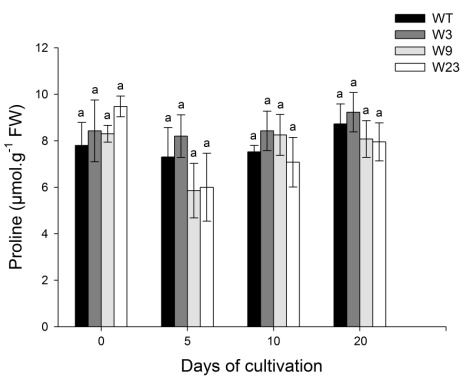

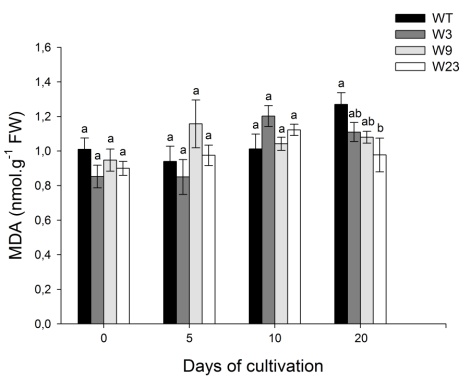

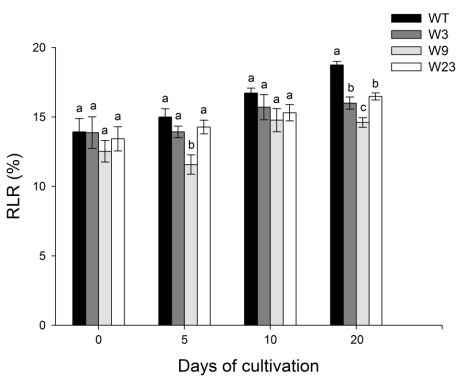


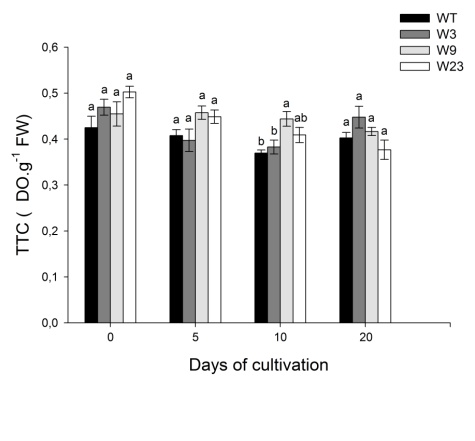


**B**

**A**

**C**

**D**

**E**

**Figure S4 |** Evaluation of oxidative-stress related parameters in *35S*::*SlWRKY3* (W3, W9 and W23) and WT lines grown under control conditions. A) Osmotic potential of Leaf 5. B) Proline and C) Malonyldialdehyde (MDA) contents of Leaf 5. D) Relative leakage rate (RLR) and E) 2,3,5-triphenyltetrazolium chloride (TTC) staining measured on Leaf 5.

Data represent means ± SEs of four biological replicates. Letters indicate values that significantly differ between tomato lines according to Student-Newman-Keuls test at p<0.05.


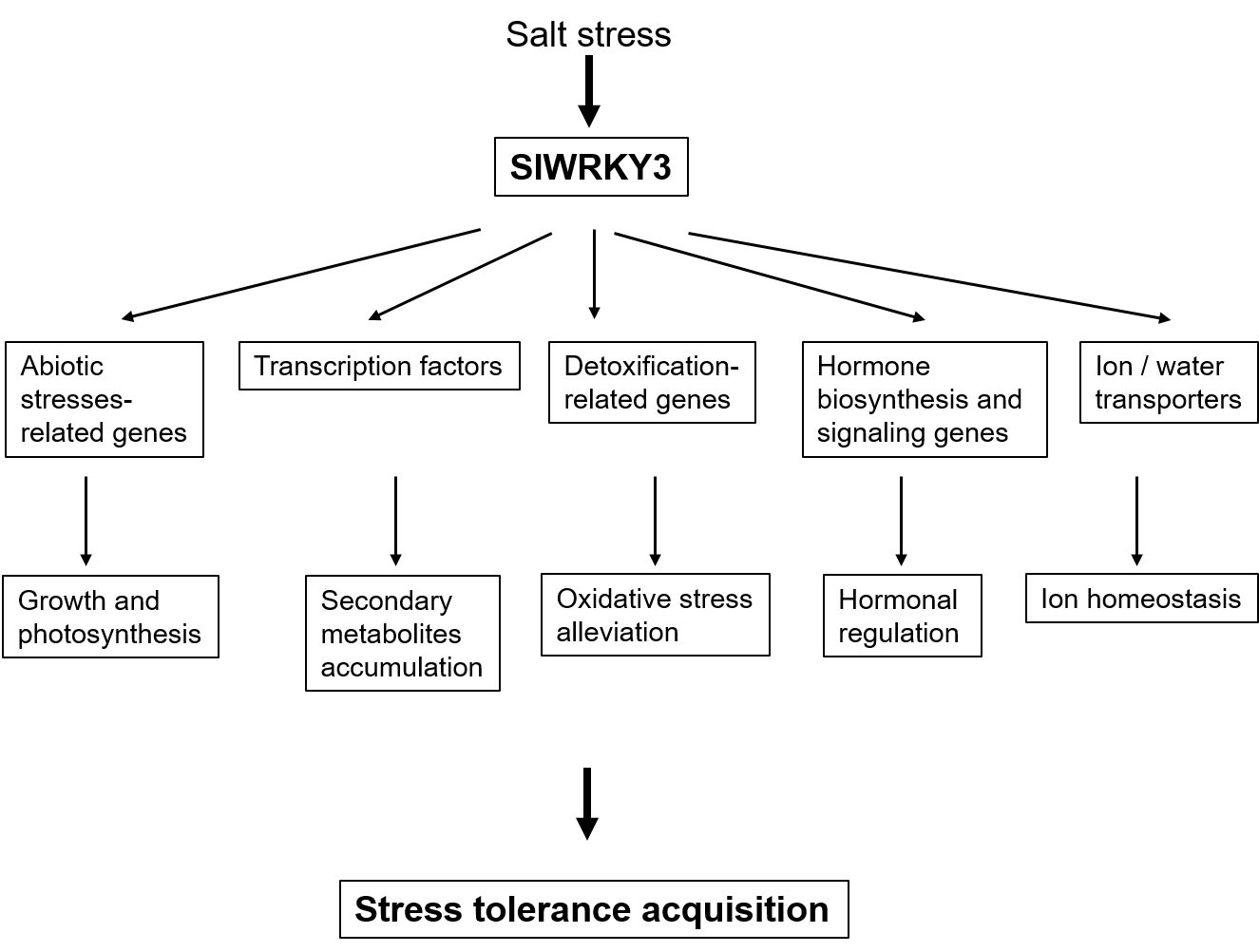


**Figure S5 |** Summary of *SlWRKY3* overexpression effects on acquisition of tolerance to salt stress in tomato.

**Table S1 |** Sequences of primers used for qRT-PCR to confirm microarray analysis.

| **Gene (accession)** | **Primers sequence (5’-3’)** | **Amplicon size (bp)** |
| --- | --- | --- |
| BaxIn (AK322997) | F: TCCATTTTTGGTGGTTCCAT  R: ACCCAAGTGAGCCTTCTCAA | 117 |
| ERF2 (AW034241) | F: TGTGTGGTGGTGCAATTCTT  R: GTTTGGGGTTGAGAGGAACA | 123 |
| LIP (AW622151) | F: CTTTTTCAGTTCGGCGATTC  R: CCGTCAGAAAAACGTCCAGT | 137 |
| MIP (BG130774) | F: GCGGAGATCATTGGAACATT  R: GGGTAGAGGCGCAAGTACAG | 105 |
| MIPS (AK321761) | F: TTGGTCCTTCTCGCTGAACT  R: GAGCCTTGGTGAGGTAGCTG | 112 |
| NAM (AK324393) | F: GAGGAATGGGAGGATGATGA  R: CGAAGGAAAAGAAGCACCAG | 150 |
| Osmotin (AK322366) | F: TTCGAGGTACGCAACAACTG  R: GACCCCATATACGTGCCATC | 136 |
| PR6 (Y08804) | F: TCCGAGAGGCCAAGCTATAA  R: AATGAACCACCATCCGTTGT | 141 |
